# Supplementary material for: Mapping the evolution of fertility support policies in China: A content and instrumental analysis
Source: PLoS One. 2025 Oct 9;20(10):e0332137. doi: 10.1371/journal.pone.0332137 (PMC12510515; doi:10.1371/journal.pone.0332137)
Supplement: S1 Appendix — (ZIP) [file pone.0332137.s001.zip › S1 Appendix. 226 original policy documents/141-中共中央、国务院印发《关于调整完善生育政策的意见》(FBM-CLI-16-215469).doc]

中共中央、国务院印发《关于调整完善生育政策的意见》

**发布部门：** [中国共产党中央委员会](https://www.pkulaw.com/law/chl?Aggs.IssueDepartment=a01) [国务院](https://www.pkulaw.com/law/chl?Aggs.IssueDepartment=5)

**发布日期：**2013.12.30

**实施日期：**2013.12.30

**时效性：** [现行有效](https://www.pkulaw.com/law/chl?Aggs.TimelinessDic=01)

**效力级别：** [党内法规制度](https://www.pkulaw.com/law/chl?Aggs.EffectivenessDic=XR12)

**法规类别：** [计划生育管理](https://www.pkulaw.com/law/chl?Aggs.Category=09705)

中共中央、国务院印发《关于调整完善生育政策的意见》
（2013年12月30日）

　　为贯彻落实党的十八届三中全会关于坚持计划生育的基本国策，启动实施一方是独生子女的夫妇可生育两个孩子的政策（以下简称单独两孩政策），逐步调整完善生育政策，促进人口长期均衡发展的决策部署，稳妥扎实有序推进调整完善生育政策，现提出如下意见。
　　一、充分认识调整完善生育政策的重要意义
　　人口问题始终是关系我国全面协调可持续发展的重大问题，是影响经济社会发展的重要因素。党中央、国务院坚持人口与发展综合决策，将计划生育确立为基本国策，积极探索符合我国实际的人口发展道路。经过全党全社会多年不懈努力，我国计划生育工作取得了巨大成就，人口过快增长得到有效控制，人口再生产类型实现历史性转变，有效缓解了人口对资源环境的压力，有力促进了经济持续较快发展和社会进步，改善了妇女儿童发展状况，为全面建成小康社会奠定了坚实基础。广大人民群众作出了重大贡献，计划生育工作者付出了艰苦努力。实践证明，从我国基本国情出发，坚定不移地推行计划生育，既符合国家长远发展要求，又符合群众根本利益，是完全正确的，必须长期坚持。
　　生育政策是计划生育基本国策的核心。现行生育政策是在长期实践中逐步形成的，并随着人口与经济社会发展形势变化不断完善。在全面建成小康社会进程中，根据人口形势发展变化，调整完善生育政策，具有十分重要的意义。
　　（一）有利于经济持续健康发展。目前，我国人口低生育水平稳中有降，15至59岁劳动年龄人口总量开始减少，劳动力平均年龄不断提高。人口老龄化速度加快，高龄化趋势明显。人口发展周期较长，在劳动力数量较丰富、人口抚养负担较轻的时期，及时调整完善生育政策，有利于改善人口结构，保持合理劳动力规模，延缓人口老龄化速度，增强经济发展活力。
　　（二）有利于家庭幸福与社会和谐。近年来，我国家庭呈现规模小型化、结构多样化趋势，独生子女家庭和独居老人比例有所升高，家庭的生育、养老等基本功能有所弱化。调整完善生育政策，顺应了人民群众期待，有利于促进出生人口性别平衡，提升家庭发展能力，有利于将当前利益与长远利益统一起来，促进社会和谐稳定。
　　（三）有利于促进人口长期均衡发展。上世纪90年代初，我国进入低生育水平国家行列。随着经济社会发展、城镇化水平提高和群众生育观念转变，妇女总和生育率呈现稳中有降的趋势。生育水平过高或过低，都不利于人口与经济社会协调发展。调整完善生育政策，符合人口发展规律，有利于稳定适度低生育水平，减缓人口总量在达到峰值后过快下降的势头，有利于中华民族长远发展。

　　二、正确把握调整完善生育政策的总体思路
　　（一）指导思想。以邓小平理论、“三个代表”重要思想、科学发展观为指导，全面贯彻党的十八大和十八届三中全会精神，坚持计划生育基本国策，贯彻实施[人口与计划生育法](https://www.pkulaw.com/chl/252f7ce51132230bbdfb.html)，稳妥扎实有序调整完善生育政策，稳定适度低生育水平，保证人口总量控制在既定规划目标之内。
　　（二）基本原则。一是总体稳定，确保政策实施过程风险可控，确保生育水平不出现大的波动。二是城乡统筹，在城乡同步调整完善生育政策，促进城乡一体化和区域协调发展。三是分类指导，在国家统一指导下，各地从实际出发作出安排。四是协调发展，统筹人口数量、素质、结构、分布的均衡发展，统筹人口与经济、社会、资源、环境的协调与可持续发展。
　　（三）方法步骤。根据[人口与计划生育法](https://www.pkulaw.com/chl/252f7ce51132230bbdfb.html)的规定，各省（自治区、直辖市）人民政府在全面评估当地人口形势、计划生育工作基础及政策实施风险的情况下，制定单独两孩政策实施方案，报国务院主管部门备案，由省级人民代表大会或其常委会修订地方性法规或作出规定，依法组织实施。

　　三、坚持计划生育基本国策，稳妥扎实有序推进各项工作
　　我国人口众多的基本国情没有根本改变，人口对经济、社会、资源、环境的压力长期存在，计划生育基本国策必须长期坚持。在调整完善生育政策过程中，必须坚持党政一把手亲自抓、负总责，必须坚持巩固和加强计划生育基层基础工作，必须坚持行之有效的工作思路和方法。
　　（一）健全工作机制。完善计划生育宣传倡导、依法管理、群众自治、优质服务、政策推动、综合治理长效工作机制，推进诚信计生工作。发挥人口计划的调控作用，建立人口监测和预警机制，加强人口变动情况调查，完善出生人口信息报告制度，加快推进国家人口基础信息库建设，实现婚姻、生育、户籍管理等方面的信息共享。继续做好重点地区特别是农村计划生育工作，做好再生育审批，倡导合理的生育间隔。加强对流动人口等重点人群的计划生育服务与管理，综合治理出生人口性别比偏高问题，严格控制政策外生育特别是多孩生育，严肃查处违法生育行为。
　　（二）完善配套政策。建立重大经济社会政策人口影响评估机制，做好相关经济社会政策与计划生育政策的有效衔接。进一步完善农村计划生育家庭奖励扶助、西部地区“少生快富”、计划生育家庭特别扶助等利益导向政策，实行奖励扶助标准动态调整机制。建立和完善计划生育家庭老年人扶助制度。妥善解决计划生育特殊困难家庭的生活照料、养老保障、大病治疗、精神慰藉等问题。做好政策调整前后计划生育利益导向和社会制约政策的衔接，维护政策的严肃性。根据人口变动情况，合理规划和配置卫生、教育、文化、社会保障等基本公共服务资源。
　　（三）做好宣传引导。深入细致地做好干部群众思想工作，把思想认识统一到中央决策部署上来。充分发挥广播电视、报刊、网络等媒体的作用，深入开展群众喜闻乐见的宣传倡导活动，大力宣传计划生育工作的巨大成就和新形势下坚持计划生育基本国策、调整完善生育政策的重要意义。加强正面引导，做好政策解读，主动回应社会关切，为政策实施营造良好舆论氛围。
　　（四）加强组织领导。各地要按照中央决策部署，加强统筹协调，确保调整完善生育政策稳妥扎实有序实施。坚持计划生育目标管理责任制，稳定和加强基层计划生育工作网络和队伍，严格落实计划生育工作一票否决制，确保责任到位、措施到位、投入到位。国务院主管部门要加强对地方调整完善生育政策工作的指导。各有关部门要增强大局观念，加强协调配合，形成工作合力。
　　各地要将本意见贯彻落实情况及时向中央报告，中央办公厅、国务院办公厅将适时组织督促检查。

© 北大法宝：（[www.pkulaw.com](https://www.pkulaw.com/)) 专业提供法律信息、法学知识和法律软件领域各类解决方案。北大法宝为您提供丰富的参考资料，正式引用法规条文时请与标准文本核对。
欢迎查看所有[产品和服务](https://www.pkulaw.com/helps/2.html)。法宝快讯：[如何快速找到您需要的检索结果？](https://www.pkulaw.com/helps/70.html)[法宝](https://www.pkulaw.com/helps/69.html)V6有何新特色？

扫描二维码阅读原文

**原文链接：**<https://www.pkulaw.com/chl/9de206735e4ad6c1bdfb.html>
